# Light Transmission and Preference of Eye Patches for Occlusion Treatment

**DOI:** 10.1371/journal.pone.0068079

**Published:** 2013-06-25

**Authors:** Hwan Heo, Jung Won Park, Sang Woo Park

**Affiliations:** Department of Ophthalmology, Chonnam National University Medical School and Hospital, Gwangju, Korea; Medical University Graz, Austria

## Abstract

**Purpose:**

To investigate light transmission and preference for six eye patches for occlusion therapy.

**Methods:**

Six patches were examined, including; Ortopad Fun Pack, Ortopad Flesh, Kawamoto A-1, Kawamoto A-2, 3M Opticlude, and Everade Eye Guard. The size and the presence of a light blocking pad of patches were investigated. The amount of light transmitted through the patches was evaluated, using a digital light meter and a model eye, in three different environments; indoors with fluorescent light, outdoors on a sunny day, and strong light from illuminator. After patching the normal eye, the flash visual evoked potential (VEP) was measured. Thirty patients with amblyopia or horizontal strabismus, who received occlusion therapy as initial treatment, were included. After using all six patches, patients completed a 7-item questionnaire regarding the patch preference for size, color and shape, adhesive power, pain with removal, skin irritation after removing patch, parent’s preference and overall opinion.

**Results:**

All patches had a light-blocking pad, except the 3M Nexcare. Ortopad had the strongest light blocking power in the three environments, and the 3M Nexcare had the weakest power. In flash VEP, Ortopad and Kawamoto patches showed flat, but 3M Nexcare and Everade Eye Guard showed normal response. There were significant preferential differences among the patches in all the items of the questionnaire (P<0.05). In comparison between the patches respectively, 3M Nexcare received the lowest satisfaction in pain when removing a patch and skin irritation after removing a patch. Kawamoto A-2 received the lowest score in the overall satisfaction.

**Conclusions:**

We found differences in the light-blocking power and in the preference of the various patches for the occlusion treatment. This is a pilot study regarding only characteristics and preferences of patches. Further clinical studies regarding the relationship between characteristics or preferences of patches and outcomes of occlusion treatment are needed.

## Introduction

Amblyopia is the most frequent cause of visual impairment in children, with a reported prevalence of 1% to 5%. [1] Patching of the non-amblyopic eye has been the mainstay of amblyopia treatment.[2]–[4] Also, patching is used in intermittent exotropia and esotropia as an antisuppression exercise to improve the fusion and reduce the angle of deviation. [5], [6] Compliance is the most important factor for effective patching treatment.[7]–[9] Many factors, such as patient’s age, initial visual acuity, refractive error and parental understanding, contribute to the compliance of eye patching. [10], [11] Nowadays, many brands of patches are available. However, there have been few investigations concerning the patch as a tool for occlusion therapy. We thought that the light transmission of the patches is variable, and cosmetic problem as well as comfort of use of the patches will affect the preference of the patches and the compliance of the patching treatment.

The purpose of this pilot study is to investigate the amount of light transmission and the preference of various eye patches for the occlusion treatment.

## Materials and Methods

### Patches and Participants

The six most popular patches of four brands were used, at the time of the study, in Korea: Ortopad Fun Pack (Ortopad, USA), Ortopad Flesh (Ortopad, USA), Kawamoto A-1 (Kawamoto, Japan), Kawamoto A-2 (Kawamoto, Japan), Nexcare (3M, USA), and Eye Guard (Everade, Korea). Between December 2009 and January 2010, the study prospectively included 32 children with newly diagnosed as amblyopia, esotropia or intermittent exotropia, with fixation preference, whose ages ranged from 3 years to 10 years, and received occlusion therapy as the initial treatment. Patients with neurologic abnormalities that prevent communication with parents or doctors were excluded. This study was approved by the Chonnam National University Hospital institutional review board. Written informed consent by the parents was a prerequisite for participation. The research adhered to the tenets of the Declaration of Helsinki.

### Evaluation of Light Transmission

**Figure 1:**
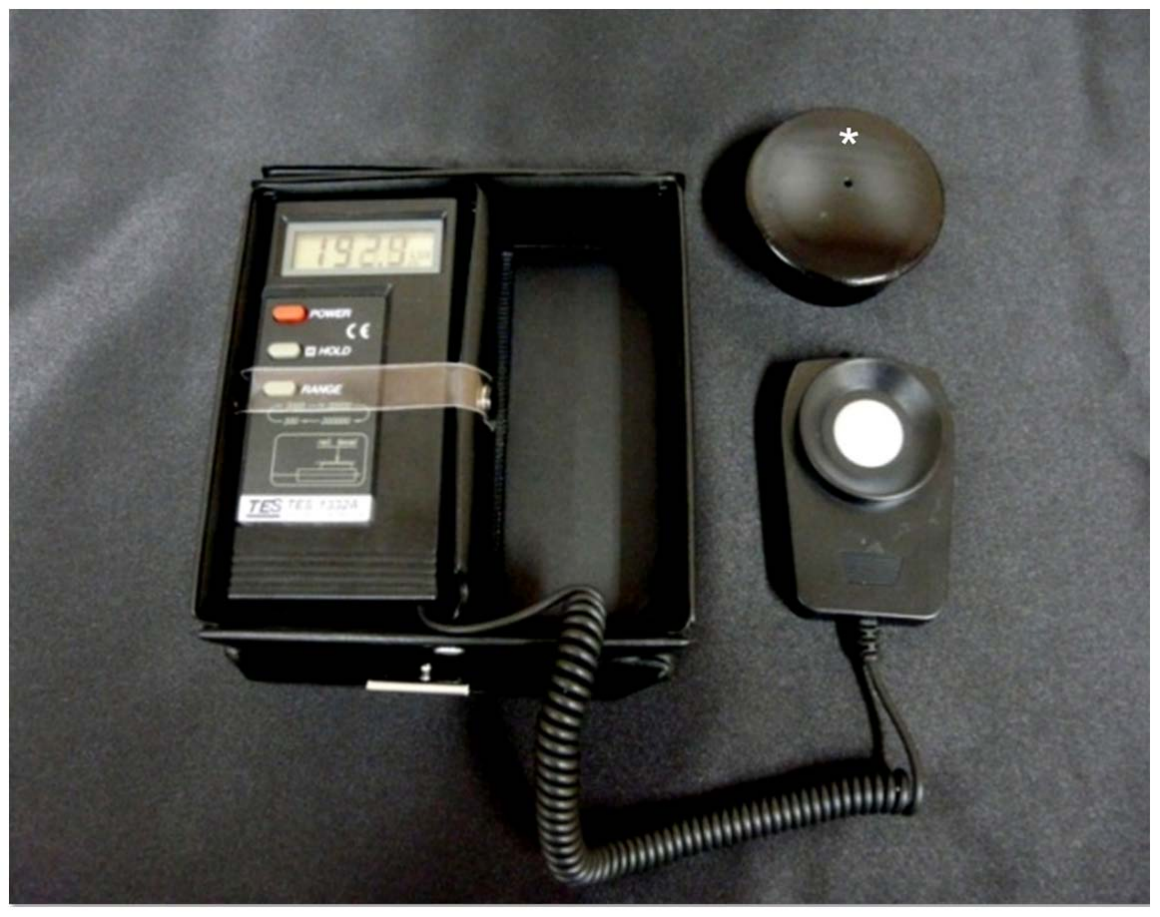
The TES Digital Light Meter and the model eye (asterisk) that can completely covered the photo sensor of light meter.

The size of the patches and pads, and the presence of a light blocking pad were investigated. We use the TES Digital Light Meter (TES Electrical Electronic Corp, Taipei) for measuring the amount of light transmitted through the patches. Spectral sensitivity of the TES Digital Light Meter was close to CIE photo pic Curve. Further, we made a model eye that has a hole with a diameter of 3.5 mm, like the pupil and the axial length of 22 mm from the hole to the photo sensor of a light meter, and that can entirely cover the photo sensor (Figure 1). After each patch covered the hole of the model eye, the transmitted light was measured in three environments; indoor with fluorescent light (one meter below the fluorescent light - about 1,000 lux) -, outdoor on a sunny day (one meter above the ground - about 10,000 lux), and strong light from an illuminator (1 centimeter from light source - about 30,000 lux).

To find out that the light transmitted through the patches can stimulate the visual pathway, we performed a flash visual evoked potential (VEP) (about 1,000 lux), according to a standard method described by the International Society for Classification of Electrophysiology of Vision, using the Medelec Synergy system (Carefusion Manufacturing, Ireland) with a goggle after patching the normal eye in two normal children (6 and 7 years old, males) with each patch, respectively.

### Preference Survey

Six patches in a separate packet identified A, B, C, D, E, and F and a questionnaire regarding preferences were provided to all patients. Each patches in a separate packet identified were given. The patch was supposed to be worn for 2 hours per day, during a week for each patch. We didn’t give any information regarding the name and manufacture of all the patches. For each patch, seven items were measured on a five-point scale satisfaction scoring system (1– strongly disagree, 2– disagree, 3– neither agree nor disagree, 4–agree, 5- strongly agree) (Table S1). Parents were instructed to fill out 1 section at the end of each week. Using this questionnaire, we compared the satisfaction on size, color, shape and adhesive power of the patches, and pain with removal, skin reaction after removing patch, parent’s preference and their overall opinion. Six weeks later, the parents were instructed to fill out the questionnaire and submit it by mail or by visiting the clinic.

### Statistical Analysis

Statistical analysis was performed using a commercially available statistical package (SPSS version 12.0 for Windows; SPSS Sciences, Chicago, USA). Data concerning the preference to patches was collected via a questionnaire. Kruskal-Willis test and Mann-Whitney U test were employed for analysis of the preference to each patch, by 7 questions. We also used a Wilcoxon rank sum test for a comparison of the preference according to sex and group activity. In all statistical analyses, a *p*<0.05 was considered significant.

## Results

### Evaluation of Light Transmission

The size of the patches and pads, and the presence of a light blocking pad were summarized in Table 1. Kawamoto A-2 was the largest, and Everade Eye Guard was the smallest. All patches had a light blocking pad, except in 3M Nexcare.

**Table 1:** The size of patches and pads, and the presence of a light blocking pad of six patches.

	Ortopad Fun Pack	Ortopad Flesh	Kawamoto A-1	Kawamoto A-2	3M Nexcare	Everade Eye guard
Size of the patch (length×width) (mm)	64×52	64×52	77×54	87×73	63×47	60×48
Size of the pad (length×width) (mm)	39×28	39×28	63×40	68×51	38×30	36×26
Light blocking pad	+	+	+	+	−	+

Table 2 shows the amount of transmitted light through each patch in a different environment, using a model eye and digital light meter. Ortopad had the strongest light blocking power in three environments, and 3M Nexcare had the weakest, which didn’t have a light-blocking pad.

**Table 2:** The amount of light transmitted through the patches.

	Ortopad Fun Pack	Ortopad Flesh	Kawamoto A-1	Kawamoto A-2	3M Nexcare	Everade Eye guard
Indoor with fluorescent light(about 1,000 lux) (lux)	0.1	0.1	0.2	0.2	1.2	0.1
Outdoor on a sunny day(about 10,000 lux) (lux)	0.1	0.1	1.5	1.5	30.9	2.1
Strong light from illuminator(about 30,000 lux) (lux)	0.1	0.1	2.2	2.2	97.4	3.2

**Figure 2:**
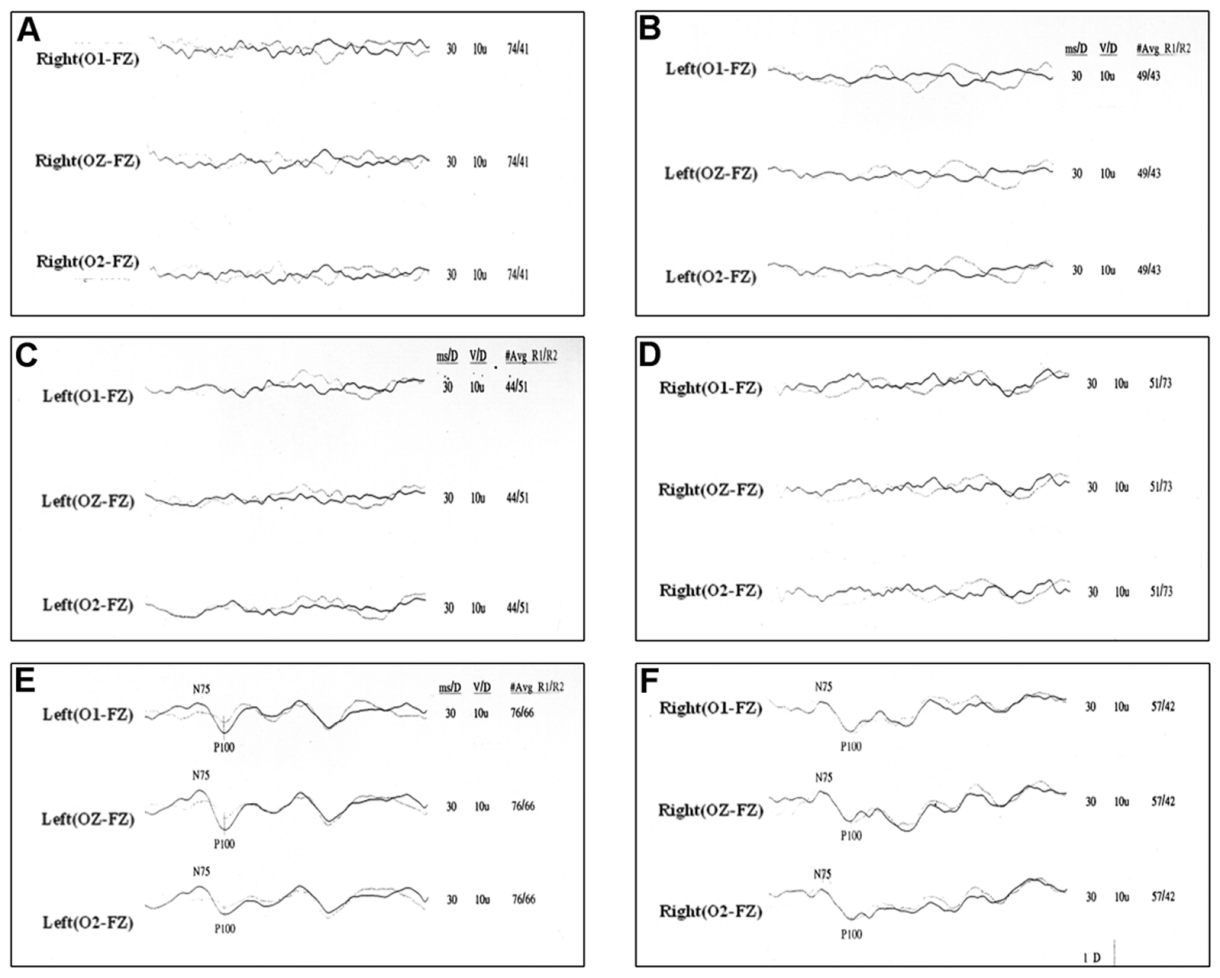
The results of flash visual evoked potential in normal eye with occlusion patch. (A) Ortopad Fun Pack (B) Ortopad Flesh (C) Kawamoto A-1 (D) Kawamoto A-2 (E) 3M Nexcare (F) Everade Eye Guard. (A) – (D) show flat response, (E) and (F) show normal response.

In flash VEP, the eyes patched with Ortopad and Kawamoto patches showed flat wave, but, the eyes patched with 3M Nexcare and Everade Eye Guard showed a normal response. This result was the same in two children (Figure 2).

### Preference Survey

Thirty-two patients were instructed about the questionnaire. Among them, 30 patients completely answered the questionnaire and were submitted. The mean age was 5.1 years old (range, 3–10 years old). There were 12 males and 18 females. There were 17 pre-school children and 13 were in school. There were 23 patients with strabismus (esotropia –8, exotropia –15) and 15 patients with amblyopia (anisometropic amblyopia –7, strabismic amblyopia –8).

The results regarding the satisfaction of the patches are summarized in Table 3. There were significant preferential differences among the patches in all items of the questionnaire (P<0.05). In comparisons between the patches respectively, Kawamoto A-2 received the lowest satisfaction in the patch size. Further, in regards to pain when removing a patch and skin irritation after a patch removal, 3M Nexcare received the lowest satisfaction. Kawamoto A-2 received the lowest score in the overall satisfaction. Ortopad Fun received the highest preference in size, pain when removing a patch and skin irritation or flares after removing a patch, adhesive power and the overall satisfaction. However, it was not statistically significant.

**Table 3:** Patch preference results presented as a mean score.

	Ortopad Fun Pack	Ortopad Flesh	Kawamoto A-1	Kawamoto A-2	3M Nexcare	Everade Eye guard	*p*-value a
**Size**	4.11±0.96	3.32±0.82	2.61±1.20	1.36±0.73 b	4.00±0.98	3.46±1.45	<0.001
**Color and design**	2.59±1.34	3.32±0.76	2.79±0.69	2.39±0.83	3.57±0.69	3.18±0.91	<0.001
**Adhesive power**	2.96±1.37	2.68±1.17	2.61±0.99	2.46±1.10	2.71±1.30	2.67±0.79	0.003
**Pain when removing patch**	4.07±0.81	3.79±0.89	3.75±0.89	3.57±0.88	1.61±0.79^b^	2.71±0.90	<0.001
**Skin irritation after removing patch**	4.44±0.79	4.21±0.88	4.07±0.94	4.11±0.92	2.61±1.20^b^	4.11±0.79	<0.001
**Child’s preference**	2.89±1.20	3.07±0.81	2.64±0.91	2.21±1.03	3.18±1.06	2.71±0.98	0.007
**Overall satisfaction**	3.04±1.64	2.71±1.01	2.46±0.96	1.60±0.69^b^	2.82±0.86	2.71±0.85	<0.001

a Kruskal-Waillis test,

b p<0.05 by Mann-Whitney U test.

In the overall satisfaction, there were no significant differences between boys and girls, and between children in preschool and in school.

## Discussion

Patching of the non-amblyopic eye is highly effective and is the most commonly used method of the treatment for amblyopia. Also, patching is used in strabismus as a non- surgical treatment. [5], [6] Many studies have demonstrated that treatment compliance is the most important factor for predicting a successful outcome in amblyopia.[10], [12]–[15] In an attempt to improve compliance, optical and pharmacological penalization were introduced and used.[16]–[18] A translucent tape on the lens and Bangerter filters were investigated. [19], [20] Also, education of parent with written information has been shown to change their attitude, and consequently, significantly increase the adherence to the treatment. [21], [22].

Until now, there were few investigations concerning the patch as a tool for occlusion therapy. Recently, Roefs AM et al. [23] reported that the comfort of wear and material properties of the eye patches were largely different. Our study focused on the amount of light blocking power, and the parent’s and children’s preferences of the patches. 3M Nexcare patch didn’t have a light blocking pad and couldn’t block the light completely in all three environments. Others all blocked the light almost completely, in all three environments.

We used the flash VEP to know whether the transmitted light through the patches can stimulate the visual system. The eye patched with 3M Nexcare and Everade Eye Guard showed normal response in flash VEP. It shows that 3M Nexcare did not have the light-blocking pad and it transmitted enough light to stimulate the visual system. The Everade Eye Guard, which has a light blocking pad, also showed normal response in flash VEP. We assumed that the small size of the light blocking pad, allowed transmission of enough light to stimulate the visual system. With the Everade Eye Guard, these unmatched results between the light transmission test using model eye and VEP test may have resulted from the limitation of our model eye, which is described below. These results showed that the transmitted lights through the some patches could stimulate the visual system in a non-amblyopic eye. We don’t know how the transmitted light through the patch affect the outcome of the occlusion treatment. However, atropine penalization has been an alternative to occlusion therapy, for the blurring vision in a sound eye for near activities, hence, forcing the amblyopic eye to be used preferentially for near vision tasks.[24]–[26] Atropine instilled eye is dilated, and more lights reach the retina than the eye with patching without a light-blocking pad. If so, the minimal transmitted light to patch might not affect the outcomes of an occlusion therapy. If we use the patches for the removing visual stimuli to the non-amblyopic eye, it is reasonable to use the patch that blocks the light completely.

In the questionnaire concerning the patch preferences, it was statistically different in all the items. Ortopad Fun Pack received the highest preference in the size of a patch, pain when removing a patch and skin irritation and overall satisfaction. Kawamoto A-2 was developed for covering the eyebrow, but its large size gave the patch the lowest preference score. 3M Nexcare received the lowest preferences score in the pain when removing a patch and skin irritation.

In Korea, 3M patch has been most commonly used, because it is very uncommon for markets to sell the other patches. As such, it has not been easy for parents to choose and buy the other patches. We thought that we have to try to introduce many kinds of patches, and therefore, the parents and children were offered a broader range of patch choices. We think that these efforts will increase the compliance of patching.

There are some limitations in this study. First, this study is pilot study and considers only characteristics and preferences of patches. Further clinical studies regarding the relationship between patch characteristics, including light transmission and patient preference, are needed to determine how these parameters affect patching treatment outcomes. Second, many manufacturers have produced various patches and improved the material and function of the patches. However, all patches could not be included in this study. Third, we did not investigate patch costs which could vary by location and may influence patch selection. Finally, the model eye used in this study did not have eyelids or orbital tissue. Therefore, patches were applied directly to the pupil, which obviously differs from the clinical situation where the patch is typically applied to the orbital adnexa or orbital rim. In a child, some light could still get through the adhesive part of the patch and enter the pupil.

This is the first pilot study to examine transmitted light through patches and preferences of patches. Our study provides insight into the difference of the light blocking power and the preference of the patches.

## Supporting Information

Table S1 **Eye Patch Questionnaire.**
(DOC)

## References

[1] Attebo K, Mitchell P, Cumming R, Smith W, Jolly N, et al (1998) Prevalence and causes of amblyopia in an adult population. Ophthalmology 105: 154–159. 9442792 10.1016/s0161-6420(98)91862-0

[2] Simons K (2005) Amblyopia characterization, treatment, and prophylaxis. Surv Ophthalmol 50: 123–166. 15749306 10.1016/j.survophthal.2004.12.005

[3] Kowal L (2002) PEDIG study on amblyopia; vision therapy by atropine penalization versus occlusion. Binocul Vis Strabismus Q 17: 275. 12470288

[4] Wallace DK, Edwards AR, Cotter SA, Beck RW, Arnold RW, et al (2006) A randomized trial to evaluate 2 hours of daily patching for strabismic and anisometropic amblyopia in children. Ophthalmology 113: 904–912. 16751033 10.1016/j.ophtha.2006.01.069 PMC1609192

[5] Spoor DK, Hiles DA (1979) Occlusion therapy for exodeviations occurring in infants and young children. Ophthalmology 86: 2152–2157. 555806 10.1016/s0161-6420(79)35295-2

[6] Figueira EC, Hing S (2006) Intermittent exotropia: comparison of treatments. Clin Experiment Ophthalmol 34: 245–251. 16671905 10.1111/j.1442-9071.2006.01199.x

[7] Simons K, Preslan M (1999) Natural history of amblyopia untreated owing to lack of compliance. Br J Ophthalmol 83: 582–587. 10216059 10.1136/bjo.83.5.582 PMC1723047

[8] Hiscox F, Strong N, Thompson JR, Minshull C, Woodruff G (1992) Occlusion for amblyopia: a comprehensive survey of outcome. Eye (Lond) 6 (Pt 3): 300–304. 10.1038/eye.1992.59 1446765

[9] Loudon SE, Polling JR, Simonsz HJ (2002) A preliminary report about the relation between visual acuity increase and compliance in patching therapy for amblyopia. Strabismus 10: 79–82. 12221485 10.1076/stra.10.2.79.8143

[10] Nucci P, Alfarano R, Piantanida A, Brancato R (1992) Compliance in antiamblyopia occlusion therapy. Acta Ophthalmol (Copenh) 70: 128–131. 1557966 10.1111/j.1755-3768.1992.tb02104.x

[11] Oliver M, Neumann R, Chaimovitch Y, Gotesman N, Shimshoni M (1986) Compliance and results of treatment for amblyopia in children more than 8 years old. Am J Ophthalmol 102: 340–345. 3752199 10.1016/0002-9394(86)90008-5

[12] Woodruff G, Hiscox F, Thompson JR, Smith LK (1994) Factors affecting the outcome of children treated for amblyopia. Eye (Lond) 8 (Pt 6): 627–631. 10.1038/eye.1994.157 7867817

[13] Fielder AR, Irwin M, Auld R, Cocker KD, Jones HS, et al (1995) Compliance in amblyopia therapy: objective monitoring of occlusion. Br J Ophthalmol 79: 585–589. 7626576 10.1136/bjo.79.6.585 PMC505171

[14] Smith LK, Thompson JR, Woodruff G, Hiscox F (1995) Factors affecting treatment compliance in amblyopia. J Pediatr Ophthalmol Strabismus 32: 98–101. 7629678 10.3928/0191-3913-19950301-09

[15] Beardsell R, Clarke S, Hill M (1999) Outcome of occlusion treatment for amblyopia. J Pediatr Ophthalmol Strabismus 36: 19–24. 9972510 10.3928/0191-3913-19990101-05

[16] France TD, France LW (1999) Optical penalization can improve vision after occlusion treatment. J AAPOS 3: 341–343. 10613577 10.1016/s1091-8531(99)70042-x

[17] Repka MX, Ray JM (1993) The efficacy of optical and pharmacological penalization. Ophthalmology 100: 769–774; discussion 774–765. 10.1016/s0161-6420(93)31577-0 8493022

[18] Foley-Nolan A, McCann A, O’Keefe M (1997) Atropine penalisation versus occlusion as the primary treatment for amblyopia. Br J Ophthalmol 81: 54–57. 9135409 10.1136/bjo.81.1.54 PMC1721990

[19] Rutstein RP, Quinn GE, Lazar EL, Beck RW, Bonsall DJ, et al.. (2010) A randomized trial comparing Bangerter filters and patching for the treatment of moderate amblyopia in children. Ophthalmology 117: 998–1004 e1006. 10.1016/j.ophtha.2009.10.014 PMC2864338 20163869

[20] Beneish RG, Polomeno RC, Flanders ME, Koenekoop RK (2009) Optimal compliance for amblyopia therapy: occlusion with a translucent tape on the lens. Can J Ophthalmol 44: 523–528. 19789586 10.3129/i09-122

[21] Goransson A, Dahlgren LO, Lennerstrand G (1998) Changes in conceptions of meaning, effects and treatment of amblyopia. A phenomenographic analysis of interview data from parents of amblyopic children. Patient Educ Couns 34: 213–225. 9791525 10.1016/s0738-3991(97)00111-0

[22] Cleary M (2000) Efficacy of occlusion for strabismic amblyopia: can an optimal duration be identified? Br J Ophthalmol 84: 572–578. 10837378 10.1136/bjo.84.6.572 PMC1723515

[23] Roefs AM, Tjiam AM, Looman CW, Simonsz-Toth B, Fronius M, et al (2012) Comfort of wear and material properties of eye patches for amblyopia treatment and the influence on compliance. Strabismus 20: 3–10. 22390325 10.3109/09273972.2012.655837

[24] Repka MX, Wallace DK, Beck RW, Kraker RT, Birch EE, et al (2005) Two-year follow-up of a 6-month randomized trial of atropine vs patching for treatment of moderate amblyopia in children. Arch Ophthalmol 123: 149–157. 15710809 10.1001/archopht.123.2.149

[25] Scheiman MM, Hertle RW, Kraker RT, Beck RW, Birch EE, et al (2008) Patching vs atropine to treat amblyopia in children aged 7 to 12 years: a randomized trial. Arch Ophthalmol 126: 1634–1642. 19064841 10.1001/archophthalmol.2008.107 PMC2846774

[26] Repka MX, Kraker RT, Beck RW, Birch E, Cotter SA, et al (2009) Treatment of severe amblyopia with weekend atropine: results from 2 randomized clinical trials. J AAPOS 13: 258–263. 19541265 10.1016/j.jaapos.2009.03.002 PMC2713117

