# Supplementary material for: Light Transmission and Preference of Eye Patches for Occlusion Treatment
Source: PLoS One. 2013 Jun 25;8(6):e68079. doi: 10.1371/journal.pone.0068079 (PMC3692472; doi:10.1371/journal.pone.0068079)
Supplement: Table S1 — Eye Patch Questionnaire. (DOC) [file pone.0068079.s001.doc]

**No.______________**

**Eye Patch Questionnaire**

ID:

Patient’s name:

| **A** | **B** |
| --- | --- |
| 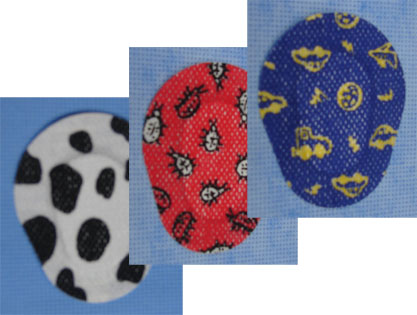 | 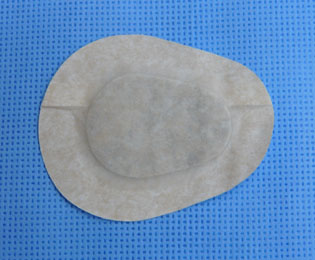 |
| **1. My child is satisfied with the size of this patch. ( )**  **(1) Strongly disagree (2) Disagree (3) Neither Agree nor Disagree (4) Agree (5) Strongly agree**  **2. My child is satisfied with the color and shape of this patch. ( )**  **(1) Strongly disagree (2) Disagree (3) Neither Agree nor Disagree (4) Agree (5) Strongly agree**  **3. My child is satisfied with the adhesive strength of this patch. ( )**  **(1) Strongly disagree (2) Disagree (3) Neither Agree nor Disagree (4) Agree (5) Strongly agree**  **4. My child didn’t feel pain when removing this patch from the skin. ( )**  **(1) Strongly disagree (2) Disagree (3) Neither Agree nor Disagree (4) Agree (5) Strongly agree**  **5. After removing this patch from the skin, there were no skin irritation and flares. ( )**  **(1) Strongly disagree (2) Disagree (3) Neither Agree nor Disagree (4) Agree (5) Strongly agree**  **6. Parents like this patch. ( )**  **(1) Strongly disagree (2) Disagree (3) Neither Agree nor Disagree (4) Agree (5) Strongly agree**  **7. Overall, my child is satisfied with this patch. ( )**  **(1) Strongly disagree (2) Disagree (3) Neither Agree nor Disagree (4) Agree (5) Strongly agree** | **1. My child is satisfied with the size of this patch. ( )**  **(1) Strongly disagree (2) Disagree (3) Neither Agree nor Disagree (4) Agree (5) Strongly agree**  **2. My child is satisfied with the color and shape of this patch. ( )**  **(1) Strongly disagree (2) Disagree (3) Neither Agree nor Disagree (4) Agree (5) Strongly agree**  **3. My child is satisfied with the adhesive strength of this patch. ( )**  **(1) Strongly disagree (2) Disagree (3) Neither Agree nor Disagree (4) Agree (5) Strongly agree**  **4. My child didn’t feel pain when removing this patch from the skin. ( )**  **(1) Strongly disagree (2) Disagree (3) Neither Agree nor Disagree (4) Agree (5) Strongly agree**  **5. After removing this patch from the skin, there were no skin irritation and flares. ( )**  **(1) Strongly disagree (2) Disagree (3) Neither Agree nor Disagree (4) Agree (5) Strongly agree**  **6. Parents like this patch. ( )**  **(1) Strongly disagree (2) Disagree (3) Neither Agree nor Disagree (4) Agree (5) Strongly agree**  **7. Overall, my child is satisfied with this patch. ( )**  **(1) Strongly disagree (2) Disagree (3) Neither Agree nor Disagree (4) Agree (5) Strongly agree** |

| **C** | **D** |
| --- | --- |
| 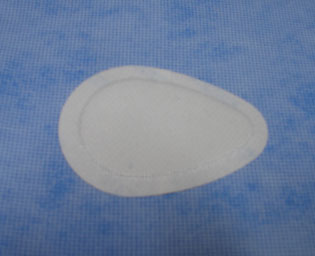 | 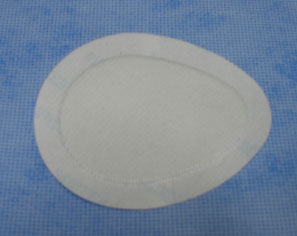 |
| **1. My child is satisfied with the size of this patch. ( )**  **(1) Strongly disagree (2) Disagree (3) Neither Agree nor Disagree (4) Agree (5) Strongly agree**  **2. My child is satisfied with the color and shape of this patch. ( )**  **(1) Strongly disagree (2) Disagree (3) Neither Agree nor Disagree (4) Agree (5) Strongly agree**  **3. My child is satisfied with the adhesive strength of this patch. ( )**  **(1) Strongly disagree (2) Disagree (3) Neither Agree nor Disagree (4) Agree (5) Strongly agree**  **4. My child didn’t feel pain when removing this patch from the skin. ( )**  **(1) Strongly disagree (2) Disagree (3) Neither Agree nor Disagree (4) Agree (5) Strongly agree**  **5. After removing this patch from the skin, there were no skin irritation and flares. ( )**  **(1) Strongly disagree (2) Disagree (3) Neither Agree nor Disagree (4) Agree (5) Strongly agree**  **6. Parents like this patch. ( )**  **(1) Strongly disagree (2) Disagree (3) Neither Agree nor Disagree (4) Agree (5) Strongly agree**  **7. Overall, my child is satisfied with this patch. ( )**  **(1) Strongly disagree (2) Disagree (3) Neither Agree nor Disagree (4) Agree (5) Strongly agree** | **1. My child is satisfied with the size of this patch. ( )**  **(1) Strongly disagree (2) Disagree (3) Neither Agree nor Disagree (4) Agree (5) Strongly agree**  **2. My child is satisfied with the color and shape of this patch. ( )**  **(1) Strongly disagree (2) Disagree (3) Neither Agree nor Disagree (4) Agree (5) Strongly agree**  **3. My child is satisfied with the adhesive strength of this patch. ( )**  **(1) Strongly disagree (2) Disagree (3) Neither Agree nor Disagree (4) Agree (5) Strongly agree**  **4. My child didn’t feel pain when removing this patch from the skin. ( )**  **(1) Strongly disagree (2) Disagree (3) Neither Agree nor Disagree (4) Agree (5) Strongly agree**  **5. After removing this patch from the skin, there were no skin irritation and flares. ( )**  **(1) Strongly disagree (2) Disagree (3) Neither Agree nor Disagree (4) Agree (5) Strongly agree**  **6. Parents like this patch. ( )**  **(1) Strongly disagree (2) Disagree (3) Neither Agree nor Disagree (4) Agree (5) Strongly agree**  **7. Overall, my child is satisfied with this patch. ( )**  **(1) Strongly disagree (2) Disagree (3) Neither Agree nor Disagree (4) Agree (5) Strongly agree** |
| **E** | **F** |
| 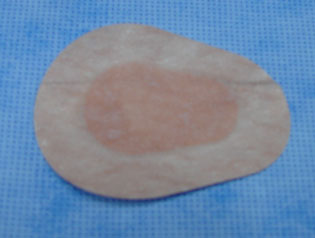 | 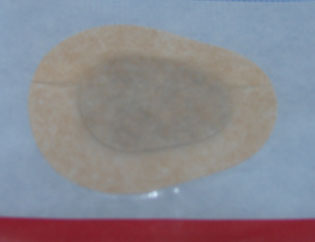 |
| **1. My child is satisfied with the size of this patch. ( )**  **(1) Strongly disagree (2) Disagree (3) Neither Agree nor Disagree (4) Agree (5) Strongly agree**  **2. My child is satisfied with the color and shape of this patch. ( )**  **(1) Strongly disagree (2) Disagree (3) Neither Agree nor Disagree (4) Agree (5) Strongly agree**  **3. My child is satisfied with the adhesive strength of this patch. ( )**  **(1) Strongly disagree (2) Disagree (3) Neither Agree nor Disagree (4) Agree (5) Strongly agree**  **4. My child didn’t feel pain when removing this patch from the skin. ( )**  **(1) Strongly disagree (2) Disagree (3) Neither Agree nor Disagree (4) Agree (5) Strongly agree**  **5. After removing this patch from the skin, there were no skin irritation and flares. ( )**  **(1) Strongly disagree (2) Disagree (3) Neither Agree nor Disagree (4) Agree (5) Strongly agree**  **6. Parents like this patch. ( )**  **(1) Strongly disagree (2) Disagree (3) Neither Agree nor Disagree (4) Agree (5) Strongly agree**  **7. Overall, my child is satisfied with this patch. ( )**  **(1) Strongly disagree (2) Disagree (3) Neither Agree nor Disagree (4) Agree (5) Strongly agree** | **1. My child is satisfied with the size of this patch. ( )**  **(1) Strongly disagree (2) Disagree (3) Neither Agree nor Disagree (4) Agree (5) Strongly agree**  **2. My child is satisfied with the color and shape of this patch. ( )**  **(1) Strongly disagree (2) Disagree (3) Neither Agree nor Disagree (4) Agree (5) Strongly agree**  **3. My child is satisfied with the adhesive strength of this patch. ( )**  **(1) Strongly disagree (2) Disagree (3) Neither Agree nor Disagree (4) Agree (5) Strongly agree**  **4. My child didn’t feel pain when removing this patch from the skin. ( )**  **(1) Strongly disagree (2) Disagree (3) Neither Agree nor Disagree (4) Agree (5) Strongly agree**  **5. After removing this patch from the skin, there were no skin irritation and flares. ( )**  **(1) Strongly disagree (2) Disagree (3) Neither Agree nor Disagree (4) Agree (5) Strongly agree**  **6. Parents like this patch. ( )**  **(1) Strongly disagree (2) Disagree (3) Neither Agree nor Disagree (4) Agree (5) Strongly agree**  **7. Overall, my child is satisfied with this patch. ( )**  **(1) Strongly disagree (2) Disagree (3) Neither Agree nor Disagree (4) Agree (5) Strongly agree** |
